# The importance of mindfulness in psychosocial distress and quality of life in dermatology patients†

**DOI:** 10.1111/bjd.14719

**Published:** 2016-09-18

**Authors:** K. Montgomery, P. Norman, A.G. Messenger, A.R. Thompson

**Affiliations:** ^1^ Department of PsychologyUniversity of SheffieldWestern BankSheffieldS10 2TPU.K.; ^2^ Department of DermatologyRoyal Hallamshire HospitalSheffieldU.K.

## Abstract

**Background:**

Mindfulness, defined as purposively and nonjudgementally paying attention in the present moment, could be used within psychosocial interventions to reduce the distress associated with social anxiety and avoidance found in many skin conditions. However, little is known about the relationship between naturally occurring levels of mindfulness and distress in dermatology patients.

**Objectives:**

To examine the relationship between mindfulness and psychosocial distress in a dermatological population. It was hypothesized that higher levels of mindfulness would be associated with lower levels of social anxiety, anxiety, depression and skin shame, and with better quality of life.

**Methods:**

Adult dermatology outpatients (*n* = 120) from one hospital completed items assessing subjective severity, skin shame, fear of negative evaluation, anxiety and depression, quality of life, and levels of mindfulness.

**Results:**

Considering depression, 14% reported mild, 5% moderate and 2·5% severe symptoms. For anxiety, 22% reported mild, 23% moderate and 6% severe symptoms. In addition, 33·4% reported clinically significant social anxiety. After controlling for subjective severity, mindfulness explained an additional 19% of the variance in depression, 39% in anxiety, 41% in social anxiety, 13% in skin shame and 6% in dermatological quality of life. One specific facet of mindfulness (acting with awareness) was found to be the most consistent predictor of distress.

**Conclusions:**

The findings indicate that higher levels of mindfulness are associated with lower distress. This suggests that facilitating mindfulness may be helpful in reducing distress in dermatology patients, and the use of mindfulness techniques warrants further investigation.

## Abstract 2

**What's already known about this topic?**

- Correlation studies suggest that higher levels of mindfulness are related to lower levels of social anxiety.
- Studies indicate that mindfulness can be beneficial for people experiencing a range of long‐term health conditions.
- Mindfulness interventions have been beneficial in improving skin clearance and severity in patients with psoriasis.

**What does this study add?**

- This is the first study to show that mindfulness accounts for a significant amount of the variance in both psychological distress and quality of life in dermatology patients.
- Mindfulness may play a particularly important role in social anxiety.
- The study suggests that facilitating the ‘ability to attend to the present moment’ is a potential target for psychosocial interventions for patients distressed by their skin condition.

**What are the clinical implications of this work?**

- High levels of social anxiety, depression and anxiety highlight the need for psychological interventions to be available to dermatology patients.
- Mindfulness interventions could benefit people living with skin conditions who are experiencing psychological distress – either as stand‐alone low‐intensity interventions or incorporated into cognitive behavioural therapy.
- Using mindfulness to bring attention to the present moment may reduce self‐focused attention, which is a key characteristic of social anxiety.

**Linked Comment:** Fordham. *Br J Dermatol* 2016; **175**:864–865.

Plain language summary available online

Video Commentary

Dermatological conditions can result in disability levels equivalent to those of other chronic diseases, affecting self‐esteem, work and relationships.^1^ It is estimated that for at least one‐third of dermatology patients effective management requires the management of psychosocial distress,^2^ with 85% of patients indicating that such factors are an important aspect of living with a skin condition.^3^ Higher rates of depression and anxiety have been reported by dermatological cohorts in comparison with the general population.^4^, ^5^ For example, people with psoriasis have reported high levels of psychosocial disability and worrying about their appearance.^6^ Similarly, high rates of anxiety have been found in people with psoriasis and atopic eczema.^7^, ^8^ Patients with acne have been found to experience depression,^2^ social anxiety^9^ and increased thoughts of suicide.^10^ Increased levels of anxiety, depression and phobic reactions have also been reported by people living with alopecia.^11^ There is a risk that people living with a skin condition can experience distress during social situations,^12^ which can result in avoidance of social encounters.^13^

For people living with a visible skin condition there is a real risk of experiencing actual negative reactions from others.^14^, ^15^ People have reported cases of discrimination such as being asked to leave public places.^16^ Not surprisingly, high levels of self‐consciousness have been described by some people living with visible conditions, and self‐consciousness has been reported to be related to avoidant coping strategies.^15^ For example, people living with alopecia often attempt to conceal hair loss, using a variety of methods.^17^ Anxiety is known to be associated with specific forms of cognitive processing such as attentional and appraisal biases, whereby the individual is at risk of misinterpreting responses of others.^18^

Patients with psoriasis have been found to demonstrate biases towards negative self‐referential words (e.g. embarrass, ugly), reactions of others (e.g. ridicule, whisper) and condition‐specific information (e.g. itching, messy) in comparison with controls.^19^ Indeed, functional magnetic resonance imaging studies suggest that patients with psoriasis may block emotional processing when seeing faces showing disgust, as a coping strategy to protect them from stressful emotional responses.^20^ Similarly, in acne a recent eye‐tracking study demonstrated that participants exhibited an attentional bias towards acne lesions in comparison with controls.^21^

Given the prevalence of psychosocial distress in dermatology patients and the potential role of attentional and interpretative bias in maintaining negative mood states,^22^, ^23^ interventions that reduce attentional bias are likely to be beneficial. Despite the clear need, access to psychological interventions within dermatology in general is limited.^3^, ^12^ A meta‐analysis of psychological interventions identified a medium effect size (*g* = 0·54)^24^ for interventions; however, the interventions that have been tested are largely limited to simple techniques focusing on specific behavioural problems. Relatively few interventions have targeted social anxiety, despite this being a common problem reported by people living with skin conditions. The present study aims to examine the extent to which mindfulness can explain levels of distress, particularly those associated with self‐consciousness (shame and social anxiety) in people living with skin conditions.

Mindfulness is defined as ‘paying attention in a particular way: on purpose, in the present moment, non‐judgementally to the unfolding experience.’^25^ It has been proposed that mindfulness consists of five distinct facets.^26^ Observing relates to a tendency to notice internal and external sensations, and awareness concerns the extent to which individuals feel able to attend to the present moment, in contrast to ‘mind wandering’. Nonjudgement of experiences concerns the ability to take a nonevaluative stance towards thoughts; describing examines the tendency to label experiences; and finally, nonreactivity to inner experience refers to the tendency to allow thoughts to come and go without responding.

Mindfulness interventions have been used with patients living with psoriasis with promising results.^27^, ^28^ Kabat‐Zinn *et al*.^27^ reported that improved skin clearing in patients with psoriasis using mindful meditations during light treatment was a result of reductions in stress. Patients with psoriasis have also reported improved dermatological quality of life and self‐assessed psoriasis severity^28^ following mindfulness‐based cognitive therapy. However, no reduction in stress levels was observed, suggesting that stress reduction was not the mechanism of change. While there is evidence that mindfulness interventions are effective with other physical health problems, it remains unclear what the underlying mechanisms are.^29^ It is therefore important to examine the different facets of mindfulness and their relationship with distress. To date, research assessing mindfulness interventions on psychosocial distress in dermatology patients has considered only psoriasis.

This study examined the relationship between mindfulness and psychosocial distress in people living with a range of visible skin conditions. It was hypothesized that higher levels of mindfulness would be associated with lower levels of social anxiety, anxiety, depression and skin shame, and with better dermatology‐specific quality of life.

## Patients and methods

Ethical approval was gained from the National Health Service, Tyne and Wear South research ethics committee. Participants were recruited from the dermatology department of a regional teaching hospital in the U.K. The inclusion criteria were (i) age > 16 years; (ii) a visible condition such as psoriasis, eczema, dermatitis, acne or hair disorders; and (iii) having sufficient English to complete the questionnaires and provide informed consent. As the study focused on skin disease, primary psychiatric diagnoses affecting the skin (e.g. trichotillomania, delusions of parasitosis) and skin cancer were excluded. Participants meeting the inclusion criteria were invited to participate following their dermatology consultation. Participants could complete the questionnaires in the clinic or at home. Questionnaires completed in the clinic were completed independently in the waiting room and returned to reception.

### Data analysis

Firstly, data were analysed to determine clinically significant levels of anxiety, depression and social anxiety using cut‐off scores. Secondly, associations were tested between demographic variables (age, sex, ethnicity), clinical variables (type of skin condition, area of body affected, subjective severity), mindfulness and psychosocial distress (social anxiety, anxiety, depression, skin shame and dermatological quality of life) using *t*‐tests, anova or correlations as appropriate. Thirdly, hierarchical regressions were conducted to examine the amount of variance in psychosocial distress explained by the five facets of mindfulness. Demographic and clinical variables found to be associated with psychological distress were entered at step one, followed by the five facets of mindfulness at step two.

### Participants

Of the 180 patients approached, 120 agreed to participate in the research (67% response rate). Reasons for declining to participate were not collected. The participants were predominantly female and white. The most frequently reported skin conditions were psoriasis, eczema, alopecia and acne. Participants not reporting their skin condition might not yet have been provided with their diagnosis. Table 1 lists full details of the sample.

**Table 1:** Sample characteristics

Characteristic	Value
Skin condition, *n* (%)	
Eczema, dermatitis, prurigo and Netherton syndrome	22 (18·3)
Acne, rosacea and dissecting cellulitis	19 (15·8)
Psoriasis	25 (20·8)
Alopecia	20 (16·7)
Other inflammatory skin conditions	8 (6·7)
Other	17 (14·2)
Not reported	9 (7·5)
Sex, *n* (%)	
Male	35 (29·2)
Female	84 (70·0)
Not reported	1 (0·8)
Ethnicity, *n* (%)	
White	104 (86·7)
Asian or Asian British	5 (4·2)
Black or black British	1 (0·8)
Other	8 (6·7)
Not reported	2 (1·7)
Age (years), mean ± SD	45·9 ± 18·4
Age at onset (years), mean ± SD	13·6 ± 14·7

### Measures

#### Mindfulness

Mindfulness was assessed using the Five Facet Mindfulness Questionnaire.^26^ The measure comprises 39 items measuring five facets of mindfulness: observing (eight items), describing (eight items), acting with awareness (eight items), nonjudgement of inner experience (eight items) and nonreactivity to inner experience (seven items). The items are rated on five‐point Likert scales and responses to each facet are summed. The individual subscales had good reliability in this study (see Table 2).

**Table 2:** Means, SDs and internal reliabilities of the main study variables

Variable	Mean	SD	Alpha
Skin shame	69·59	17·98	0·92
Social anxiety	20·77	8·73	0·95
Depression	4·80	3·80	0·81
Anxiety	7·85	4·44	0·84
Dermatological quality of life	8·00	7·14	0·89
Describe	26·55	6·01	0·83
Observe	22·56	6·23	0·77
Awareness	29·22	6·12	0·87
Nonreactivity	20·38	6·13	0·86
Nonjudgement	29·54	6·93	0·89

#### Subjective severity

In order to examine participants’ perceptions of the severity of their skin condition, visibility and impact on daily life, participants were asked to indicate, on five‐point scales, the extent to which they felt their skin condition affected their life, how visible it was, and whether they received negative comments. Scores on these items were summed to provide a total score. Participants also reported the area of the body affected.

#### Skin shame

The Skin Shame Scale (Scott, unpublished thesis) measures an individual's personal experience of skin distress. It is based on models of shame relating to dermatology and disfigurement and consists of 24 questions rated on five‐point response scales. It has been reported to be a reliable (Cronbach α = 0·92) and valid measure.^30^, ^31^

#### Social anxiety

The Brief Fear of Negative Evaluation straightforward items^32^, ^33^, ^34^ was used to assess social anxiety. It consists of eight statements that participants are asked to rate on five‐point response scales. Cronbach α‐values for clinical, control and combined samples have been reported at 0·92, 0·90 and 0·96, respectively.^34^ A cut‐off score of 25 was used to identify individuals experiencing symptoms of social anxiety disorder.^33^

#### Anxiety and depression

The Hospital Anxiety and Depression Scale^35^ comprises seven items assessing anxiety and seven items assessing depression, rated on four‐point response scales. A score of eight or above on either scale indicates presence of anxiety or depression symptoms, with 15 or above indicating severe anxiety or depression symptoms. It has been widely used in psychiatric, primary‐care and general populations.^35^ It has good psychometric properties (Cronbach α for anxiety = 0·83 and depression = 0·80).^36^

#### Dermatological quality of life

The DLQI^37^ consists of 10 items concerning the impact of the skin condition over the last week rated on four‐point Likert scales. Items relate to symptoms and feelings, daily activity, leisure, work/school, personal relationships and side‐effects of treatment. The DLQI has good validity, reliability and responsiveness to change.^38^

## Results

### Demographic variables

Associations between age, sex, age of onset, type of skin condition and psychological outcomes were examined. Age was the only demographic variable significantly correlated with social anxiety, *r*(117) = −0·29, *P* = 0·002; dermatological quality of life, *r*(113) = −0·21, *P* = 0·025; and skin shame, *r*(116) = −0·25, *P* = 0·007.

### Psychological distress variables

Scores on outcome variables were examined to establish the prevalence of anxiety, depression and social anxiety. For depression, 77% reported no symptoms, 14% reported mild, 5% moderate and 3% severe symptoms of depression. For anxiety, 47% reported no symptoms, 22% mild, 23% moderate and 6% severe symptoms. Clinically significant levels of social anxiety were reported by 33·4% of the sample. Over half of the sample (51·7%) reported the skin condition having some effect on their quality of life, with 26·7% reporting a very large or extremely large effect.

### Subjective severity

Subjective severity was significantly positively correlated with skin shame, *r*(117) = 0·65, *P* < 0·001; depression, *r*(118) = 0·49, *P* < 0·001; anxiety, *r*(119) = 0·42, *P* < 0·001; dermatological quality of life, *r*(114) = 0·66, *P* < 0·001; and social anxiety, *r*(118) = 0·41, *P* < 0·001. Thus, higher levels of subjective severity were related to higher levels of psychosocial distress.

### Area of body affected by the skin condition

Participants who indicated that their skin condition affected their head had significantly higher levels of social anxiety, *t*(116) = 2·44, *P* = 0·03 and higher levels of depression, *t*(116) = 2·46, *P* = 0·02. Participants whose skin condition affected their arms reported significantly higher levels of depression, *t*(116) = 2·22, *P* = 0·028, and poorer dermatological quality of life, *t(*112) = 3·07, *P* = 0·003. In addition, participants whose skin condition affected their hands, *t*(112) = 4·23, *P* < 0·001; legs, *t*(112) = 3·57, *P* = 0·001; and feet, *t*(112) = 2·30, *P* = 0·024 reported significantly lower dermatological quality of life.

### Mindfulness and psychosocial distress

Correlations between mindfulness and measures of psychological distress are presented in Table 3. The results highlight different patterns of associations between individual facets of mindfulness and psychosocial distress. ‘Describe’, ‘act with awareness’ and ‘nonjudgement of inner experience’ were negatively correlated with all measures of psychological distress. Higher levels of mindfulness on these three facets were related to lower levels of skin shame, social anxiety, anxiety, depression and dermatological quality of life. In addition, ‘observe’ was positively correlated with social anxiety and anxiety. ‘Nonreactivity to inner experience’ was not significantly correlated with any of the dependent variables.

**Table 3:** Correlations between mindfulness and measures of psychological distress

	Skin shame	Social anxiety	Anxiety	Depression	Quality of life
Describe	−0·33**	−0·37**	−0·28**	−0·27**	−0·22*
Observe	0·07	0·24*	0·19*	0·13	0·12
Awareness	−0·37**	−0·64**	−0·65**	−0·46**	−0·22*
Nonreactivity	−0·05	0·03	−0·04	−0·06	−0·11
Nonjudgement	−0·35**	−0·63**	−0·53**	−0·42**	−0·25**

*P < 0·05, **P < 0·01.

A series of hierarchical regression analyses was conducted to examine the amount of variance in psychological outcomes explained by the five facets of mindfulness (Table 4). As subjective severity was significantly correlated with all of the psychological outcomes, it was controlled for in the regression analyses. The regression analyses were repeated controlling for other demographic variables correlated with specific outcomes; however, the results were unchanged. For the sake of brevity and consistency, only the analyses controlling for subjective severity are reported.

**Table 4:** Summary of regression analyses of variables predicting psychological outcomes

	Skin shame		Social anxiety		Anxiety		Depression		Quality of life	
	β	β	β	β	β	β	β	β	β	β
Step 1										
Subjective severity	0·64***	0·58***	0·44***	0·30***	0·41***	0·28***	0·45***	0·36***	0·66***	0·63***
Step 2										
Describe		−0·23**		−0·12		−0·06		−0·07		−0·14
Observe		0·02		0·03		0·09		0·08		0·19*
Awareness		−0·19*		−0·38***		−0·53*		−0·32**		−0·09
Nonreactivity		−0·001		−0·09		−0·18*		−0·13		−0·13
Nonjudgement		−0·04		−0·26***		−0·13		−0·11		0·03
Δ*R* ^2^	0·42***	0·13***	0·19***	0·41***	0·17***	0·39***	0·20***	0·19***	0·44***	0·06*

*P < 0·05, **P < 0·01, ***P < 0·001.

After controlling for subjective severity, mindfulness explained additional variance in skin shame, Δ*R* ^2^ = 0·13, Δ*F*(5,101) = 5·56, *P* < 0·001; social anxiety, Δ*R* ^2^ = 0·41, Δ*F*(5,101) = 20·09, *P* < 0·001; anxiety, Δ*R* ^2^ = 0·39, Δ*F*(5,101) = 20·09, *P* < 0·001; depression, Δ*R* ^2^ = 0·19, Δ*F*(5,100) = 6·06, *P* < 0·001; and dermatological quality of life, Δ*R* ^2^ = 0·06, Δ*F*(5,99) = 2·31, *P* = 0·05. Act awareness was the most consistent predictor and was significant in all regression analyses except for quality of life. In addition, describe was a significant predictor of skin shame, observe was a significant predictor of quality of life, and nonjudgement was a significant predictor of social anxiety.

## Discussion

High levels of anxiety, depression and social anxiety were documented in the present sample of dermatology patients, therefore adding to the literature highlighting the need for psychological support.^1^, ^2^, ^3^, ^4^, ^5^, ^6^, ^7^, ^8^, ^9^, ^10^, ^11^, ^12^, ^13^, ^14^, ^15^, ^16^, ^17^ The present results also suggest that people living with visible skin conditions who report higher levels of mindfulness report lower levels of psychosocial distress and better quality of life, across a range of different skin conditions.

Regression analyses indicated that mindfulness explained 6–41% of the variance in psychosocial distress. Higher levels of both present‐moment awareness and nonjudgement of inner experience were associated with lower levels of social anxiety. These results suggest that individuals with high levels of social anxiety are paying less attention to the present moment. Acting with awareness could reduce the tendency to engage in rumination, which has been found to maintain distress.^39^ Cognitive models of social phobia propose that social anxiety is maintained by attentional and interpretative biases.^22^, ^40^ Attention is focused on internal processes, and the individual becomes less engaged with the social situation.^22^, ^40^ Previous findings of bias towards negative self‐referential information in patients with psoriasis suggest that reducing attentional bias would be beneficial in reducing symptoms of social anxiety in people with skin conditions.

Individuals reporting higher levels of awareness also reported lower levels of depression and anxiety. With regards to depression, Teasdale *et al*.^39^ proposed that if cognitive resources are deployed in the present moment this decreases the resources available for ruminative processing. Given the role that rumination plays in maintaining other disorders,^22^ this might explain the mechanism by which acting with awareness might operate, although this supposition requires further investigation.

While mindfulness was significantly associated with quality of life, the pattern of results differed from the other psychosocial variables. ‘Observe’, the facet concerning the tendency to notice internal and external sensations, was not related in the direction expected, as higher scores related to more impairment in quality of life. This may be particularly relevant to dermatology patients, given that they may experience unpleasant physical sensations due to their condition or be using unpleasant emollients or treatments. The differential pattern of results for ‘observe’ has been found in previous studies.^41^ It may suggest that increased attention to emotions and distress in the absence of a nonjudgemental attitude could lead to an increase in distress.^41^

Participants reporting higher ‘nonreactivity to experience’ experienced lower levels of anxiety. Previous studies suggest that nonreactivity is a key component of mindfulness, given the role it plays in reducing negative automatic emotional responding.^42^ Nonreactivity may be particularly relevant for dermatology patients who experience distressing physical symptoms related to their condition, or negative thinking related to their appearance. Lower levels of awareness were related to higher levels of skin shame, suggesting that participants are acting on automatic pilot and are more likely to engage with habitual patterns of negative thinking characteristic of depressive mood states.^41^ The relationship between ‘describe’ and skin shame may suggest that individuals who are able to describe their experiences in an objective manner are likely to experience lower levels of skin shame.

Overall, the different pattern of relationships between mindfulness facets and psychosocial distress suggests that not all aspects of mindfulness may be important targets for modification in psychological interventions. Nevertheless, the results suggest that mindfulness interventions could be beneficial for people experiencing psychosocial distress and living with a visible skin condition. Current interventions used in dermatology reporting the largest effect sizes include habit reversal (*g* = 1·05) and cognitive behavioural therapy (CBT) (*g* = 0·65).^24^ CBT has been found to be effective in reducing distress associated with dermatological conditions in a small number of studies;^43^, ^44^ however, these studies have limited power due to small sample sizes. Considering social anxiety, a significant number of patients fail to reach remission following CBT.^45^ The present findings suggest that further studies examining mindfulness techniques in dermatological populations would be beneficial. Specifically, the findings suggest that individuals with social anxiety may benefit from using mindfulness to (i) bring attention to the present moment and reduce ruminative processes; and (ii) reduce the tendency of automatically judging the content of thoughts.

The current study design has a number of limitations that should be noted. Firstly, the cross‐sectional design of this study precludes strong conclusions being drawn about the direction of effects. Longitudinal and interventional studies are required to address this issue. Secondly, although studies suggest that objective severity is not an accurate indicator of distress,^46^, ^47^ as all measures used were self‐reported, future studies might seek to gain clinician ratings of severity. Thirdly, as the sample was predominantly white and female, some caution should be exhibited about the generalizability of the findings. Furthermore, this prevented an examination of sex differences in distress and mindfulness. Finally, the fact that the sample was drawn from a specialist dermatology clinic also places boundaries on the generalizability of the results.

In conclusion, the findings of this study suggest that higher levels of mindfulness, particularly awareness, are associated with reduced psychosocial distress and improved dermatological quality of life. Mindfulness explained the highest proportion of variance in social anxiety in this study, which suggests that mindfulness interventions might be used to target social anxiety associated with skin conditions. While the results should be interpreted with caution given the cross‐sectional nature of the study, the findings offer evidence that increasing mindfulness may be helpful in reducing distress in people living with visible skin conditions.

## Supporting information


**Video S1.** Author video
